# Neuroendocrine Carcinoma of Cervix: A Case Series

**DOI:** 10.7759/cureus.39165

**Published:** 2023-05-17

**Authors:** Adarsh Vardhan Tangella, Deepak C Yadlapalli

**Affiliations:** 1 Internal Medicine, Andhra Medical College and King George Hospital, Visakhapatnam, IND; 2 Medical Oncology, GSL (Ganni Subbulakshmi Garu) Cancer Trust Hospital, Rajamahendravaram, IND; 3 Medical Oncology, GSL (Ganni Subbulakshmi Garu) Medical College, Rajamahendravaram, IND

**Keywords:** case-series, gynaecologic oncology, human papillomavirus (hpv), cervical carcinoma, neuroendocrine carcinoma of cervix

## Abstract

Cervical cancer is the second most common cause of cancer-related mortality in women globally. Neuroendocrine carcinomas are among the rarest and least studied histopathological types of cervical cancers, accounting for 1.4% of all cervical cancers. Neuroendocrine carcinomas of the cervix (NECCs) are aggressive tumors that can be associated with several high-risk features such as early lymphovascular invasion and multiple systemic metastases, at early stages. Here, we present a case series of five patients with NECC who have been diagnosed and managed at a tertiary care hospital in coastal Andhra Pradesh, South India. Using the hospital records, we made a list of patients with NECC who were diagnosed by histopathological findings between 2019 and 2022. Details regarding their demographic variables, presenting complaints, staging, and treatment given were noted down using a pre-defined proforma.

## Introduction

Globally, cervical cancer is the second most common cause of cancer-related mortality in women [1]. It has already been noted that most cases of cervical cancer have a significant association with human papillomavirus (HPV) infection [2]. Neuroendocrine carcinomas of the cervix (NECCs) are extremely rare histopathological types of cervical cancers and account for about 1.4% of all cervical cancers [3]. NECCs are aggressive tumors [4] that can be associated with several high-risk features such as early lymphovascular invasion and multiple systemic metastases at early stages [3]. Poor prognosis can be attributed to two main factors: the highly malignant nature of the tumor and late diagnosis. The long-term overall survival (OS) rate of this variant is poor. Hence, it is important to diagnose it early and treat it accordingly and aggressively. Here, we present a series of five cases of NECC, which have been diagnosed and managed at a tertiary care hospital in coastal Andhra Pradesh, India.

## Case presentation

Patient 1

A 35-year-old pre-menopausal woman presented with irregular vaginal bleeding for three months and white vaginal discharge for one week. She was the mother of two children with no family history of malignancies. On vaginal examination, a 3 x 4 cm growth was felt over the anterior lip of her cervix which was friable. Her vaginal fornices were free. There was no associated inguinal lymphadenopathy. Ultrasound scans of the abdomen and pelvis showed a hypoechoic 3 x 3 cm lesion on the anterior cervical lip. MRI scans of the pelvis showed well-defined homogeneous signal intensity from a mass of 3.8 x 2.5 cm in the cervix with suspicious minimal parametrial infiltration. Histopathological examination of a cervical biopsy done in the past showed adenosquamous changes. She was scheduled for a radical hysterectomy with bilateral salpingo-oophorectomy and bilateral pelvic node dissection. The surgically retrieved specimen was sent for histopathological examination, which showed features suggestive of papillary adenocarcinoma with neuroendocrine features and was involving more than half of the thickness of the cervical stroma. There were no signs of a lymphovascular, parametrial, uterine, or upper vaginal invasion. No tumor deposits were present in the patient’s pelvic lymph nodes. Immunohistochemical (IHC) staining showed strong cytoplasmic activity with chromogranin in most tumor cells suggestive of high-grade NECC. She began treatment with concurrent etoposide and cisplatin for two cycles with radiotherapy, followed by two cycles of adjuvant cisplatin and etoposide. During her treatment, she had an episode of febrile neutropenia which was managed accordingly. The patient did not return for follow-up for more than eight months. Upon return to the clinic, she was re-admitted with etoposide and cisplatin chemotherapy for three cycles. After six months, ultrasound scans of her abdomen and pelvis showed lesions in the right and left lobes of the liver which raised suspicion of metastases. Fine-needle aspiration cytology biopsy of these lesions was advised but the patient did not report back.

Patient 2

A 50-year-old post-menopausal woman came with a complaint of vaginal bleeding. She had right upper and lower limb weakness associated with dysarthria. She was also hypertensive and non-diabetic. Upon examination, a 3 x 3.5 cm mass was seen on the uterine cervix involving the posterior fornix. Chest X-ray revealed superior mediastinal widening suggestive of a possible mediastinal mass or metastatic lymph node. Ultrasound of the abdomen showed a 3 x 2.5 cm cervical mass and bilateral mild hydronephrosis. MRI scan of the pelvis revealed a 3.7 x 2.7 x 2.5 cm mass originating from the uterine cervix involving the posterior fornix, upper one-third of the vagina, and left parametrium. Abdominal and pelvic lymph nodes on the MRI were suggestive of possible lymphatic metastases. A CT brain scan showed hypodense lesions in the bilateral frontal and parieto-occipital region and chronic infarcts in the right caudate nucleus, left internal capsule, and right cerebellar hemisphere. MRI brain scans showed infarcts in the same areas as mentioned previously. Histopathological examination of a tru-cut biopsy of the cervical mass suggested features of NEC. The patient began treatment with an etoposide and cisplatin chemotherapeutic regimen for six cycles to be given once in three weeks.

Patient 3

A 31-year-old pre-menopausal woman presented with heavy vaginal bleeding and lower abdominal pain for six months. She was a mother of two and has no family history of malignancies. On examination, her abdomen was soft and a vaginal examination showed a 4 x 5 cm ulceroproliferative mass on the cervical lip protruding into the vagina. Fluorodeoxyglucose (FDG), positron emission tomography (PET), and CT scans showed metabolically active soft tissue density lesion in the cervix along with metabolically active left pelvic, right crural, and cervical nodes. PET/CT scans also showed increased uptake in lytic lesions over the intertrochanteric region of the right femur and left iliac bone suggesting skeletal metastases. Histopathological examination of cervical biopsy was suggestive of a malignant small round cell tumor of the cervix with the possibility of NEC. Immunohistochemistry showed dot-like positivity with creatine kinase, diffuse positivity with synaptophysin, and focal positivity with chromogranin while p16 was positive and Ki67 was around 80%. These findings confirmed the diagnosis of small-cell NECC. As per the International Federation of Gynecology and Obstetrics (FIGO) classification, this grade IV disease was treated with etoposide and cisplatin for six cycles given once in three weeks along with zoledronic acid for the weakening of bones due to lytic bone metastases.

Patient 4

A 54-year-old post-menopausal woman was referred to the clinic after surgical fixation of a pathological subtrochanteric fracture of her right femur. On examination, she had an ill-defined mass in her lower abdomen and tenderness of the right femur. Ultrasound scans of the abdomen showed an ill-defined heterogeneous lesion measuring 8.2 x 7.5 cm, which involved both anterior and posterior cervical lips and caused proximal dilatation of the endometrium. Following a cervical biopsy, the histopathology was suggestive of small-cell NEC. The surgical specimen retrieved during her femoral fixation also showed similar histopathology. PET, CT, and IHC markers could not be conducted due to financial reasons. To rule out other causes of lytic bone lesions, bone marrow aspiration and serum protein electrophoresis were done. Bone marrow aspiration showed marrow that was normocellular for age with normal erythropoiesis and granulopoiesis, and megakaryocytes and plasma cells constituted 5% of nucleated bone marrow cells. Serum protein electrophoresis was suggestive of hypergammaglobulinemia with raised alpha globulins. She was diagnosed with stage IV NECC and was referred to radiotherapy for skeletal metastases. She also began palliative chemotherapy with etoposide and cisplatin for six cycles along with zoledronic acid plus calcium to treat the lytic bone lesions.

Patient 5

A 48-year-old pre-menopausal woman with three children and normal bowel and bladder habits presented with complaints of white foul-smelling discharge with menarche and lower abdominal and back ache for two years. Upon examination, a 3 x 2 cm endocervical growth was noted and the vaginal fornices, mucosa, and bilateral parametrium were free. Furthermore, the rectal mucosa was soft. A cervical biopsy showed histopathology, which correlated with NECC. The patient was diagnosed with grade IB NECC and was posted for radical hysterectomy with bilateral salpingo-oophorectomy and bilateral pelvic node dissection. Histopathological examination of the surgical specimen showed features of NEC, involving more than half of one side of the cervical stroma. Pelvic nodes and other pelvic structures were free from tumor deposits and invasion. She was started on radiotherapy and concurrent cisplatin and etoposide for two cycles given once in three weeks, and after completing radiotherapy, she was started on adjuvant cisplatin and etoposide for two more cycles given once in three weeks.

Tables 1, 2 show the summary of the above five patients.

**Table 1:** Comparison of the cases based on immunohistochemistry. NP: not performed.

	Creatine kinase	Chromogranin A	Synaptophysin	p16	Neuron-specific enolase	Ki67
Patient 1	NP	Positive	NP	NP	NP	NP
Patient 2	NP	NP	NP	NP	NP	NP
Patient 3	Positive	Positive	Positive	Positive	NP	80%
Patient 4	NP	NP	NP	NP	NP	NP
Patient 5	NP	NP	NP	NP	NP	NP

**Table 2:** Comparison of the five cases of neuroendocrine carcinoma of the cervix based on various parameters. NA: not available.

	Age (years)	Chief complaints	Duration of complaints	Parity	Chemotherapy	Surgery/radiotherapy	Site(s) of metastases (if present)	Neuroendocrine secretory features	Stage
Patient 1	35	Irregular vaginal bleeding and white discharge	3 months	2	Etoposide and cisplatin for 2 cycles with concurrent radiotherapy followed by 3 cycles of etoposide and cisplatin	Concurrent radiotherapy	Liver	No	IB at admission, progression to IV
Patient 2	50	Irregular vaginal bleeding	8 months	2	Etoposide and cisplatin for 6 cycles given once in three weeks	-	Abdominal and pelvic lymph nodes	No	IV
Patient 3	31	Irregular vaginal bleeding and lower abdominal pain	6 months	2	Etoposide and cisplatin for 6 cycles given once in 3 weeks + zoledronic acid	-	Pelvic lymph nodes, crural lymph nodes, cervical lymph nodes and femur	No	IV
Patient 4	54	Lower abdominal pain	NA	1	Etoposide and cisplatin for 6 cycles given once in 3 weeks + zoledronic acid + calcium	Radical hysterectomy with bilateral salpingo-oophorectomy and bilateral pelvic node dissection	Femur	No	IV
Patient 5	48	Lower abdominal pain and white discharge	2 years	2	Etoposide and cisplatin for 2 cycles with concurrent radiotherapy followed by 2 cycles of etoposide and cisplatin given once in three weeks	Concurrent radiotherapy	NA	No	IB

## Discussion

NECC is a rare tumor that is associated with poor prognosis and poor OS. It is an aggressive tumor associated with early vascular metastasis and poor response to treatment. Although NECC patients have high mortality rates, there are no specific guidelines about the treatment protocols or ways to measure the treatment response.

NECC commonly presents with symptoms similar to other cervical cancers, such as irregular vaginal bleeding, postcoital bleeding, or postmenopausal bleeding [5]. Sometimes, patients can present with neuroendocrine paraneoplastic symptoms such as Cushing’s syndrome, hypercalcemia, or syndrome of inappropriate antidiuretic hormone secretion [5,6]. NECC is more frequently reported in parous women and those ≥60 years old, which is a higher age than the mean age of incidence in adenocarcinoma of the cervix [5]. The diagnosis of NECC is primarily based on histopathological findings. As per the WHO guidelines, NECC can be categorized into two broad groups: well-differentiated and poorly differentiated. Well-differentiated NECC is further subclassified into three groups: NECC G1 (carcinoid), NECC G2 (atypical carcinoid), and NECC G3. Poorly differentiated can be small-cell NEC and large-cell NEC. This classification is based on histopathological findings and tumor activity. The most common histological type is small-cell NEC.

It has been proven that NECC has a strong association with HPV infection [2]. High-risk HPV strains, especially HPV 18, are the most common serotypes linked with this tumor [7,8]. This association is primarily because of the oncogenic nature of HPV, which can be attributed to the production of E6 and E7 proteins by the virus which cause disruption of cell cycle control by the dysregulation of cell cycle control genes such as the RB1 gene. Studies showed that PIK3CA is the most common gene that is mutated in these patients, followed by KRAS and GNAS [7,9]. When compared with neuroendocrine tumors of the lung or bladder, NECC has a very low incidence of TP53 mutations. The genetic mutation profile of NECC is more similar to common cervical cancer variants than small-cell neuroendocrine tumors of the lung or bladder.

The diagnosis of NECC is confirmed by immunohistochemistry. The IHC markers that are usually used are synaptophysin, chromogranin, neuron-specific enolase (NSE), creatine kinase, Ki67, and CD56. At least one marker has to be positive in order to confirm a case of NEC [5]. NSE is generally highly expressed and hence is a sensitive marker; however, the specificity of NSE is low. NSE can also be seen in the serum and can be used as a serum tumor marker sometimes [3]. Synaptophysin and chromogranin A are highly specific IHC markers for NECC but the sensitivity is low.

Once NECC is confirmed, a proper treatment plan has to be charted. FIGO classification can be used to stage the disease and plan accordingly. For early stages (<IIB), a surgical approach by radical hysterectomy and adnexectomy is advisable followed by postoperative adjuvant chemotherapy and radiotherapy [10,11]. In the advanced stages, chemotherapy and radiotherapy are advised. According to the guidelines of the Society for Gynecological Oncology (SGO), NECC tumor less than 4 cm in size needs to be surgically managed first and then followed by chemo and radiotherapy [4]. The most widely used chemotherapy regimen is etoposide with platinum-based drugs such as cisplatin. Apart from it, alternate regimens that can be used are cisplatin and irinotecan [12] or paclitaxel and carboplatin [13], or cisplatin with vincristine and bleomycin [14]. Patients with recurrent NECC who have already been treated with etoposide and cisplatin can be managed using topotecan, paclitaxel, and bevacizumab [15,16]. Apart from these conventional chemotherapeutic regimens, a few patient cases were reported on improved clinical outcomes with nivolumab [17] and trametinib [18].

As PIK3CA and KRAS mutations are common in these tumors [9], targeted therapy may potentially be effective. These tumors might also express PD-L1 which can represent a potential option for immunotherapy [8]. A study on brachytherapy along with external beam radiation therapy in NECC patients showed that brachytherapy can be associated with significant OS improvement if used in the management of FIGO II, III, and stage IVA tumors [19]. However, no significant improvements were reported in patients with FIGO I and stage IVB tumors.

Risk analysis is very essential in the case of NECC due to its extremely guarded prognosis. The mean OS is around 40 months and the five-year OS rate is around 34% [3] compared with five-year OS rates of 74.3% and 64.6% in patients with squamous cell and adenocarcinomas [20] demonstrating high morbidity and mortality of NECC. The five-year survival rate for early-stage tumors is around 30-46% and 0-15% for advanced-stage tumors, which indicates the poor prognosis of NECC [10]. Metastases can be identified even in the early stages and the rapid progression of the disease also points toward the possibility of micrometastases which should be tackled during the initial phases of the treatment [20]. The factors that are associated with poor OS are advanced FIGO stage, large tumor size, lymph nodal metastases, and depth of stromal invasion on histopathological examination (more than two-thirds of the stromal thickness).

This study has some limitations. Our hospital runs on a government-sponsored health scheme named “Aarogya Sree.” Unfortunately, immunohistochemistry is not covered by this scheme hence not allowing us to perform it for free to all patients. This is the reason why we had to base our diagnosis purely on histopathological features as only a couple of patients were financially sound to get immunohistochemistry done in a private lab.

## Conclusions

When compared to other cervical cancers, NECC is associated with higher rates of metastases, morbidity, and mortality. Its association with HPV infection suggests that it could possibly be prevented. Women, especially those between the ages of 21 and 65 years, should be advised about pap smear and HPV testing because these screening tests have been linked to early detection and a significant decline in the diseases brought on by HPV. Prompt testing, an early and accurate diagnosis, as well as vigorous treatment methods, can increase these patients' chances of OS and disease-free survival. To avoid a delay in therapy, it is crucial to be aware of this carcinoma and carefully assess the histology of a cervical biopsy.
